# Cerebral fat embolism and the "starfield" pattern: a case report

**DOI:** 10.1186/1757-1626-2-212

**Published:** 2009-11-19

**Authors:** Amit Aravapalli, James Fox, Christos Lazaridis

**Affiliations:** 1 Department of Internal Medicine, Medical University of South Carolina, 96 Jonathan Lucas Street, Charleston, SC 29425, USA; 2 Department of Neurosciences, Neurosciences Intensive Care Unit, Medical University of South Carolina, 96 Jonathan Lucas Street, Charleston, SC 29425, USA

## Abstract

Nearly all long-bone fractures are accompanied by some form of fat embolism. The rare complication of clinically significant fat embolism syndrome, however, occurs in only 0.9-2.2% of cases. The clinical triad of fat embolism syndrome consists of respiratory distress, altered mental status, and petechial rash. Cerebral fat embolism causes the neurologic involvement seen in fat embolism syndrome. A 19-year-old African-American male was admitted with gunshot wounds to his right hand and right knee. He had diffuse hyperactive deep tendon reflexes, bilateral ankle clonus and decerebrate posturing with a Glasgow Coma Scale (GCS) score of 4T. Subsequent MRI of the brain showed innumerable punctate areas of restricted diffusion consistent with "starfield" pattern. On a 10-week follow up he has a normal neurological examination and he is discharged home. Despite the severity of the neurologic insult upon initial presentation, the majority of case reports on cerebral fat embolism illustrate that cerebral dysfunction associated with cerebral fat embolism is reversible. When neurologic deterioration occurs in the non-head trauma patient, then a systemic cause such as fat emboli should be considered. We describe a patient with non-head trauma who demonstrated the classic "starfield" pattern on diffusion-weighted MRI imaging.

## Introduction

While fat embolism occurs in nearly all long-bone fractures, the incidence of clinically significant fat embolism syndrome (FES) after long-bone fractures is only 0.9% to 2.2% [1,2]. The clinical triad of FES consists of respiratory distress, altered mental status, and petechial rash. Cerebral fat embolism (CFE) consists of multiple microembolic infarcts giving a picture of a "starfield" pattern in diffusion-weighted MRI imaging (DWI) [2,3]. Neurologic symptoms can be transient and widely varied from a diffuse encephalopathy to focal deficits, all the way to neurological devastation and death. Though often non-fatal, patients typically require extended hospital stays for supportive care as their symptoms resolve [4]. We describe a patient with non-head trauma who presented with coma and demonstrated the "starfield" pattern on (DWI).

## Case presentation

**Figure 1:**
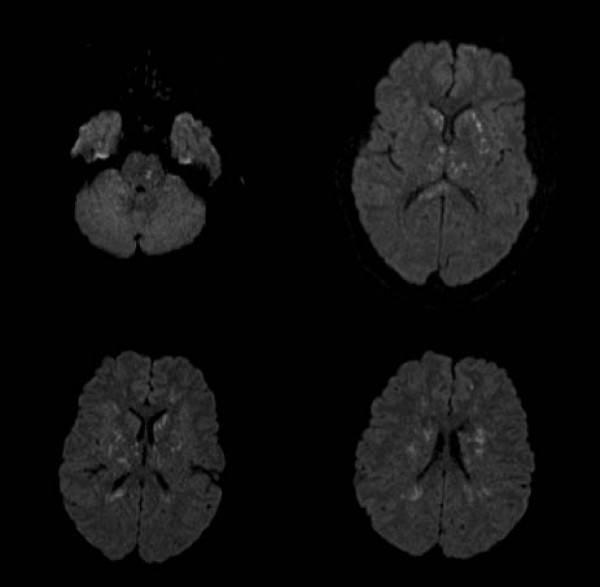
**MRI shows punctate areas of restricted diffusion within the basal ganglia, thalami, splenium of the corpus callosum, deep white matter, frontoparietal cortex and pons**.

A 19-year-old African-American male without past medical history was admitted with gunshot wounds to his right hand and right knee. He was intubated upon arrival secondary to generalized tonic-clonic seizure activity. He was found to have an open proximal phalanx fracture of the right ring finger and an open right lateral femoral condyle fracture. Initial CT of the head was unremarkable for intracranial abnormalities, and continuous EEG ruled out non-convulsive status epilepticus. He was taken to the operating room for debridement and immobilization of both open fractures and then admitted to our intensive care unit (ICU). On neurological exam, patient was unarousable with eyes closed and spontaneous blinking. His pupils were dilated but responsive to light. Petechiae were observed in the nasal aspect of the left eye. He had normal occulocephalic, corneal, and cough reflexes. He had diffuse hyperactive deep tendon reflexes, bilateral ankle clonus and decerebrate posturing with a Glasgow Coma Scale (GCS) score of 4T. Subsequent MRI of the brain showed innumerable punctate areas of restricted diffusion within the basal ganglia, thalami, splenium of the corpus callosum, deep white matter, frontoparietal cortex and pons (Figure 1). CT angiogram of the head and neck showed patency of major cervical and intracranial vessels and no evidence of vasculitis. Urine toxicology was positive for methamphetamine and cannabis. Transthoracic echocardiography showed no cardiac abnormalities and revealed no embolic source. On a 10-week follow up he has a normal neurological examination and he is discharged home.

## Discussion

The outcome of patients with FES who receive supportive care generally leads to resolution of pulmonary, neurological, and dermatological involvement with mortality of less than 10%. The majority of case reports on CFE illustrate that cerebral dysfunction associated with CFE is reversible [2-4]. Ryu et al. described findings on DWI on six patients with CFE secondary to large bone fractures. Level of consciousness in these series varied from drowsiness to coma. The DWI images showed hyperintense dot-like lesions disseminated in the brain consistent with a "starfield" pattern and Fluid-Attenuated Inversion Recovery (FLAIR) sequence MRI showed confluent hyperintense lesions in the white matter. The lesions were distributed mainly but not exclusively in the bilateral border-zone areas. Five out of six patients had either full neurological recovery or mild disability within ten days of admission [3]. Gregorakos et al. describe two cases involving teenage males suffering from prolonged coma secondary to CFE and lower extremity fractures from motor vehicle collisions (MVC). MRI showed multiple areas of increased signal intensity in the cerebral white matter in the first patient and diffuse high signal intensity in periventricular and subcortical white matter in the second one. Despite prolonged coma for 40 days, both of these patients completely recovered and their repeat MRI showed resolution of lesions [4]. Parizel et al. describe a teenage female who fractured her left tibia secondary to MVC. MRI T2 weighted images, two days after the accident, showed punctate foci of high signal intensity in subcortical white and gray matter (basal ganglia and thalami). DWI showed a "starfield" pattern. This patient had full neurological recovery by one week and repeat MRI four weeks post MVC revealed disappearance of signal abnormalities [2].

The majority of CFE cases have reversible sequela. There is a correlation between the MRI findings of CFE patients and their clinical manifestations according to Takayashi et al. Cases with good outcome had complete resolution of high-intensity lesions on subsequent MRI studies [5]. DWI should be performed in non-head trauma patients who either present or develop altered mental status, as CFE needs to be considered in these patients. The "starfield" pattern is commonly associated with good prognosis despite severity of the neurologic insult upon presentation.

## Consent

We've made repeated efforts to reach the patient and his family in order to obtain consent. Unfortunately we were not able to contact them. There is no patient identifiable information in our text or images and there is no reason to think that the patient or their family would object to publication.

## Competing interests

The authors declare that they have no competing interests.

## Authors' contributions

All authors participated in the patient's treatment. AA, JF, and CL were all major contributors in writing the manuscript. All authors read and approved the final manuscript.
